# Mechanisms of allergen-specific immunotherapy and immune tolerance to allergens

**DOI:** 10.1186/s40413-015-0063-2

**Published:** 2015-05-14

**Authors:** Cezmi A Akdis, Mübeccel Akdis

**Affiliations:** Swiss Institute of Allergy and Asthma Research (SIAF), Obere Strasse 22, CH7270 Davos, Switzerland

## Abstract

Substantial progress in understanding mechanisms of immune regulation in allergy, asthma, autoimmune diseases, tumors, organ transplantation and chronic infections has led to a variety of targeted therapeutic approaches. Allergen-specific immunotherapy (AIT) has been used for 100 years as a desensitizing therapy for allergic diseases and represents the potentially curative and specific way of treatment. The mechanisms by which allergen-AIT has its mechanisms of action include the very early desensitization effects, modulation of T- and B-cell responses and related antibody isotypes as well as inhibition of migration of eosinophils, basophils and mast cells to tissues and release of their mediators. Regulatory T cells (Treg) have been identified as key regulators of immunological processes in peripheral tolerance to allergens. Skewing of allergen-specific effector T cells to a regulatory phenotype appears as a key event in the development of healthy immune response to allergens and successful outcome in AIT. Naturally occurring FoxP3^+^ CD4^+^CD25^+^ Treg cells and inducible type 1 Treg (Tr1) cells contribute to the control of allergen-specific immune responses in several major ways, which can be summarized as suppression of dendritic cells that support the generation of effector T cells; suppression of effector Th1, Th2 and Th17 cells; suppression of allergen-specific IgE, and induction of IgG4; suppression of mast cells, basophils and eosinophils and suppression of effector T cell migration to tissues. New strategies for immune intervention will likely include targeting of the molecular mechanisms of allergen tolerance and reciprocal regulation of effector and regulatory T cell subsets.

## Introduction

The immune system forms an interactive network with tissues and makes it’s decisions on the basis of signals coming from resident tissue cells, infectious agents, commensal bacteria and almost any environmental agents. Our research during the last years has focused on different aspects for the development of novel concepts on how the immune system tolerates allergens, and how allergic diseases should be redefined accordingly [1-29]. In recent years, induction of immune tolerance has become a prime target for prevention and treatment strategies for many diseases in which dysregulation of the immune system plays an important role [30]. Currently, allergen-specific immunotherapy (AIT) is mainly applied subcutaneously or sublingually and is suitable for both children and adults for pollen, pet dander, house dust mite, and venom allergies [31-34]. It not only affects rhinoconjunctival symptoms but also has documented short- and long-term benefits in asthma treatment. The disease modification effects of AIT leads to decreased disease severity, less drug usage, prevention of future allergen sensitizations, and a long-term curative effect. Increasing safety while maintaining or even augmenting efficiency is the main goal of research for novel vaccine development and improvement of treatment schemes in AIT [32-34].

Immune tolerance to allergens can be defined as establishment of a long-term clinical tolerance against allergens, which immunologically implies changes in memory type allergen-specific T and B cell responses as well as mast cells and basophil activation thresholds that do not cause allergic symptoms anymore [35-39]. In addition, prevention of new allergen sensitizations [40] and progression to more severe disease, such as development of asthma [41] after allergic rhinitis or development of systemic anaphylaxis are main clinical implications of immune tolerance [42-46]. Many different ways of treatments are being pursued to improve efficacy, decrease side effects, decrease long course of treatment and increase patient compliance [47-51]. Currently pursued novel approaches are epicutaneous AIT and combination of peptides of grass pollen allergens with hepatitis B virus Pre S protein and peptide immunotherapy with short and long T cell epitope petides and intralymphatic immunotherapy [52-55]. Studies to provide prophylactic usage are also being performed [56]. Many efforts are being performed for the improvement and standardization of conventional subcutaneous and sublingual AITs as well as oral immunotherapy of food allergy from patient selection, to vaccine applications and treatment schedules [57-63].

The immunologic basis of allergic diseases is observed in two phases: sensitization and development of memory T and B cell responses and IgE (early phase), and effector functions related to tissue inflammation and injury (late phase) [37]. The differentiation and clonal expansion of allergen-specific CD4^+^ Th2 cells producing IL-4 and IL-13 are essential to induce class switching to the ε immunoglobulin heavy chain in B cells and the production of allergen-specific IgE antibodies during the sensitization phase. Allergen-specific IgE binds to the high-affinity FcεRI on the surface of mast cells and basophils, thus leading to the patient’s sensitization [64]. When a new encounter with the allergen causes cross-linking of the IgE-FcεRI complexes on sensitized basophils and mast cells, they are activated and subsequently release of anaphylactogenic mediators responsible for the classical symptoms of the immediate phase (type 1 hypersensitivity).

Depending on the innate immune response activating capacity of the substances co-exposed with the antigen, co-signals for cell differentiation and status of the cells and cytokines in the microenvironment, CD4^+^ naive T cells can differentiate into Th1, Th2, Th9, Th17 or Th22 type memory and effector cells. Based on their respective cytokine profiles, responses to chemokines and interactions with other cells, these T-cell subsets can promote different types of inflammatory responses. During the development of allergic disease, effector Th2 cells produce IL-4, IL-5, IL-9, IL-13 [35-37,65,66] and probably other recently identified cytokines such as IL-25, IL-31, IL-33 mainly secreted from epithelial cells and dendritic cells contribute to Th2 responses [67-73]. These cytokines play a role in the production of allergen-specific IgE, eosinophilia, permissiveness of endothelium for the recruitment of inflammatory cells to inflamed tissues, production of mucus and decreased threshold of contraction of smooth muscles [74]. The commonly observed Th2 profile in atopic diseases might be a result of a) increased differentiation and clonal expansion of Th2 cells [75] or b) increased tendency to activation-induced cell death of high IFN-γ-producing Th1 cells [76]. Th1 cells also efficiently contribute to the effector phase in allergic diseases with their role in apoptosis of the epithelium in asthma and atopic dermatitis [77-79], and apoptosis of smooth muscle cells in fatal asthma [80].

The discovery of the Th17 cells is filling an essential gap in our understanding of inflammatory processes. Th17 cells are characterized by IL-17A, IL-17 F, IL-6, IL-8, TNF-α, IL-22 and IL-26 expression [81-87]. Neutralization of IL-17 and Th17-related functions resolves tissue pathology in autoimmunity models, reduces joint destruction in experimental arthritis and reduces neutrophil infiltration in an experimental asthma model, while increasing eosinophil infiltration [88-91]. It was shown in two recent studies that TGF-β in the presence of IL-4 reprograms Th2 cell differentiation and leads to the development of a new population of Th9 cells that produce IL-9 and IL-10 [92,93].

T cell subset known as Th22 cells has been demonstrated in T cells that independently express IL-22 with low expression levels of IL-17 and play a role in atopic dermatitis [94]. All these T subsets and related events represent targets in the treatment of allergic diseases and the induction of Treg cells and allergen tolerance can balance their over activation.

The pivotal role of Treg cells in inducing and maintaining immune tolerance has been demonstrated during the last 15 years, where their adoptive transfer was shown to prevent or cure several T-cell mediated disease models, including asthmatic lung inflammation, autoimmune diseases and allograft rejection [95]. In the clinical setting, both injection and sublingual versions of AIT have been shown to induce allergen-specific Treg cells in humans. In addition to Treg cells, several other factors appear to play a mechanistic role in AIT. It is now essential to identify biomarkers and predict the response to AIT. Novel understanding of disease endotypes will help to further develop this concept [96]. Novel developments in molecular biology such as microRNAs may provide novel targets [97]. miRNAs function together with partner proteins and mainly cause gene silencing through degradation of target mRNAs or inhibition of translation. A particular miRNA can have hundreds of target genes including interleukins and their co expressed pro or anti-inflammatory genes, and thereby influence the expression of a large proportion of proteins [97-100].

### Molecular and cellular events in AIT and their underlying mechanisms

#### Very early mast cell and basophil suppression-related desensitization effect

**Figure 1:**
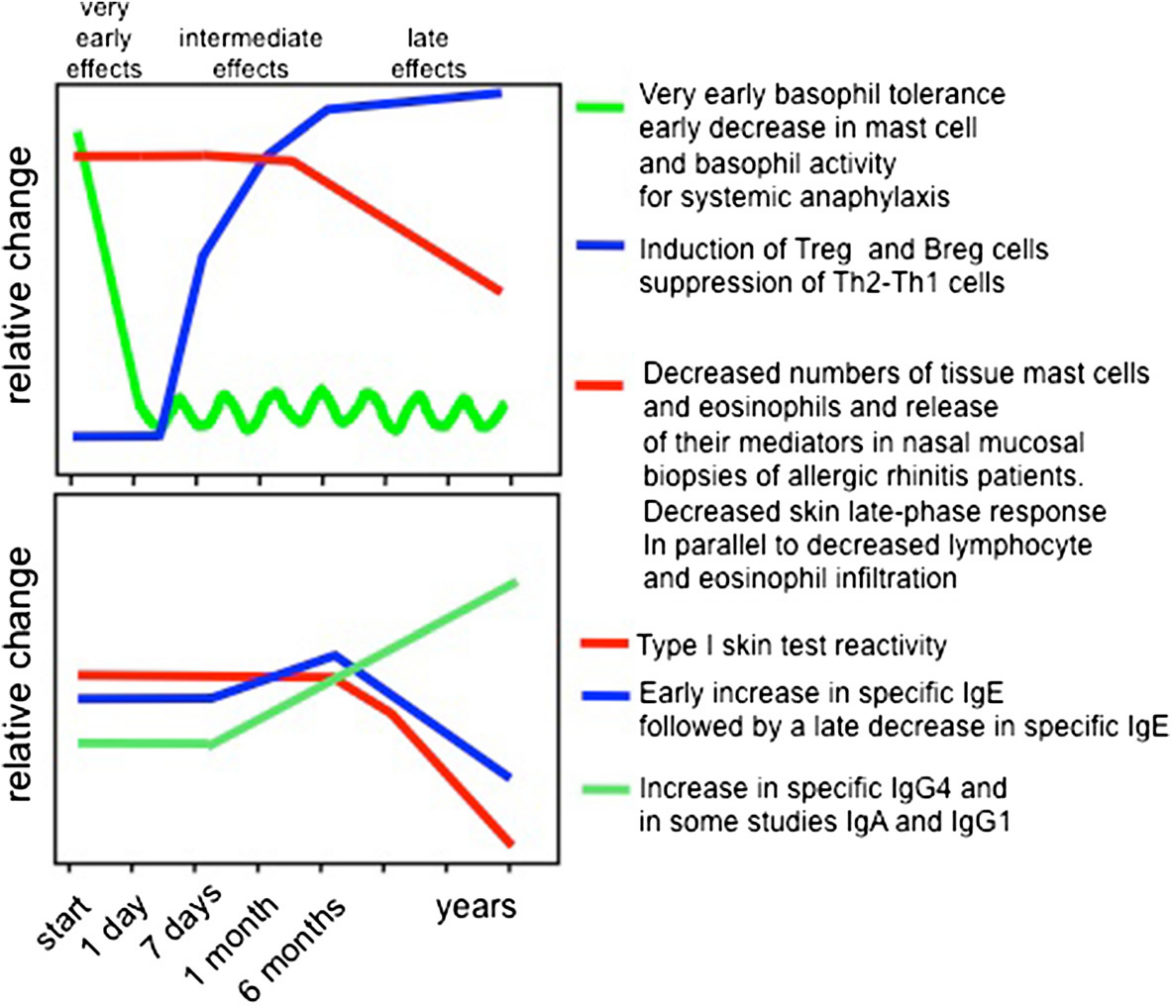
**Immunologic changes during the course of AIT.** Starting with the first injection, decreases in mast cell and basophil activity, degranulation and tendency for systemic anaphylaxis degranulation takes place within the first hours. This is followed by generation of allergen-specific Treg and Breg cells and suppression of allergen-specific Th1 and Th2 cells. Specific IgE shows an early increase and decreases relatively late. These events are in parallel to increases of IgG4 that continuously increases as long as the treatment continues. After several months, the allergen-specific IgE/IgG4 ratio decreases. After a few months, decreases in tissue mast cells and eosinophils and release of their mediators and skin late phase response occurs. A significant decrease in type I skin test reactivity is also observed relatively late in the course. It has to be noted that there is significant variation between donors and protocols.

Although decreases in IgE antibody levels and IgE-mediated skin sensitivity normally requires years of AIT, most patients are protected against bee stings or tolerate skin late phase response challenges at early stages of respective venom or grass pollen SITs [101,102]. An important observation starting from the first injection is an early decrease in mast cell and basophil activity for degranulation and systemic anaphylaxis (Figure 1). There is surprisingly little information about the mechanisms by which AIT modifies and/or suppresses immune responses of basophils and mast cells, in particular during repetitive administration of increasing doses of allergens within the first hours. Although it seems similar to rapid desensitization for hypersensitivity reactions to drugs, the mechanism of this desensitization effect for AIT is yet unknown. Acute oral desensitization in mice demonstrated that antigen-specific mast cell desensitization is one of the main underlying mechanisms for oral desensitization [103]. It has been shown that mediators of anaphylaxis (histamine and leukotrienes) are released during AIT and sting challenges without inducing a systemic anaphylactic response [104]. Their piecemeal release below the threshold of systemic anaphylaxis may decrease the granule content of mediators and also may affect the threshold of activation of mast cells and basophils, because decreased mediator release in these cells is a well demonstrated feature a short time after the start of AIT [104-106]. One of the main soluble factors liberated by effector cells following allergen challenge is histamine, which mediates its effects via histamine receptors (HRs). So far, four different human HR-types have been identified as H1-4 [107]. Both the expression pattern of HRs and modifications in the intensity of the expression of a single HR type are decisive for the nature of the developing immune response [108,109]. H1R has significant proinflammatory and cell activating properties, while H2R has been shown to be coupled to adenylate cyclase and phosphoinositide second messenger systems and is supposed to be involved primarily in tolerogenic immune responses [110]. Although there are individual differences and risks for developing systemic anaphylaxis during the course of AIT, the suppression of mast cells and basophils continues to be affected by changes in other immune parameters such as the generation of allergen-specific Treg cells and decreased specific IgE. In a recent study, significantly enhanced tryptophan degradation and elevated human Ig receptors inhibitory transcript (ILT4) expression in monocytes were found within a few hours after the first injection on day 1 representing markers of very early changes [111]. In addition, early improvement in basophil sensitivity predicts symptom relief with grass pollen immunotherapy [112]. Furthermore, basophil expression of diamine oxidase shows a significant increase after AIT and suggested as a novel biomarker of allergen immunotherapy response [113].

#### Very early effects related to antigen-presenting cells and adjuvants

Aluminium hydroxide is a commonly used adjuvant in AIT vaccines. While generally proven to be efficacious and having a good safety profile, novel adjuvants are needed to overcome current problems in conventional immunotherapy. For example depending on the type of toll-like receptor (TLR), different types of antigen-presenting cells can be targeted. TLR-triggering compounds that can control the overexpression of Th2 cytokines or skew the Th1-Th2 balance towards a Th1 and Treg profile have been effective in murine models of allergy [114].

The epidermis contains high numbers of potent antigen-presenting Langerhans cells. Accordingly, transcutaneous or epicutaneous AIT was recently introduced as a treatment option for allergies [115]. A few applications of allergens using skin patches with treatment duration of a few weeks were sufficient to achieve lasting relief. Similarly oral mucosal Langerhans cells bind allergens after resorbtion, which significantly increased their migratory capacity but attenuated their maturation [116]. Allergen challenge promoted the release of TGF-beta1 and IL-10 by oral mucosal Langerhans cells themselves as well as by cocultured T cells.

The tolerogenic function of different types of DC depends on certain maturation stages and subsets of different ontogenies and can be influenced by immunomodulatory agents. A role for DC in the induction of different subsets of Treg cells in defined microenvironments has been supported by several studies. In intestinal lamina propria, several subsets of DC reside and are in close contact with commensal bacteria and food antigens/allergens [117,118]. DC from the lamina propria of the small intestine and from the mesenteric lymph node are noticeably better than splenic DC at inducing the expression of Foxp3 in naive T cells in the presence of exogenous TGF-β [117,118]. Treg cells can be induced in the microenvironment of tumors and chronic infections due to DC that promote them. In some cases, DC conditioned by Foxp3^+^ Treg cells; pathogen-derived molecules such as, filamentous hemagglutinin [119]; exogenous signals such as histamine via its receptor 2 [110], adenosine [120], Vitamin D3 metabolites [121] or retinoic acid [122] can induce new populations of Treg cells. Antigen presentation by partially mature airway DC that express IL-10 induce the formation of Tr1-like cells, which inhibit subsequent inflammatory responses [123]. In addition, depletion and adoptively transfer of pulmonary plasmocytoid DC has demonstrated an important role for these cells in protection from allergen sensitization and asthma development in mice [124].

Virus-like particles as a novel, modular, acellular antigen-presenting system and as strong adjuvants are able to modulate the responses of allergen-specific T cells. Displaying Fel d1 on virus-like particles prevents type I hypersensitivity despite greatly enhanced immunogenicity and represents a novel therapy for cat allergy. A single vaccination was sufficient to induce protection in mice [125,126].

Innate lymphoid cells are a recently introduced cell subset that may play a role in enhancing inflammation in many diseases. Particularly Type 2 innate lymphoid cells play a role in asthma and upper respiratory inflammation [127]. Type 2 immunity consists of GATA-3+ ILC2s, TC2 cells, and Th2 cells producing IL-4, IL-5, and IL-13, which induce mast cell, basophil, and eosinophil activation, as well as IgE antibody production, thus protecting against helminthes and venoms [128]. Seasonal increases in peripheral innate lymphoid type 2 cells are inhibited by subcutaneous grass pollen immunotherapy [129].

#### Treg cells and peripheral T cell tolerance to allergens

**Figure 2:**
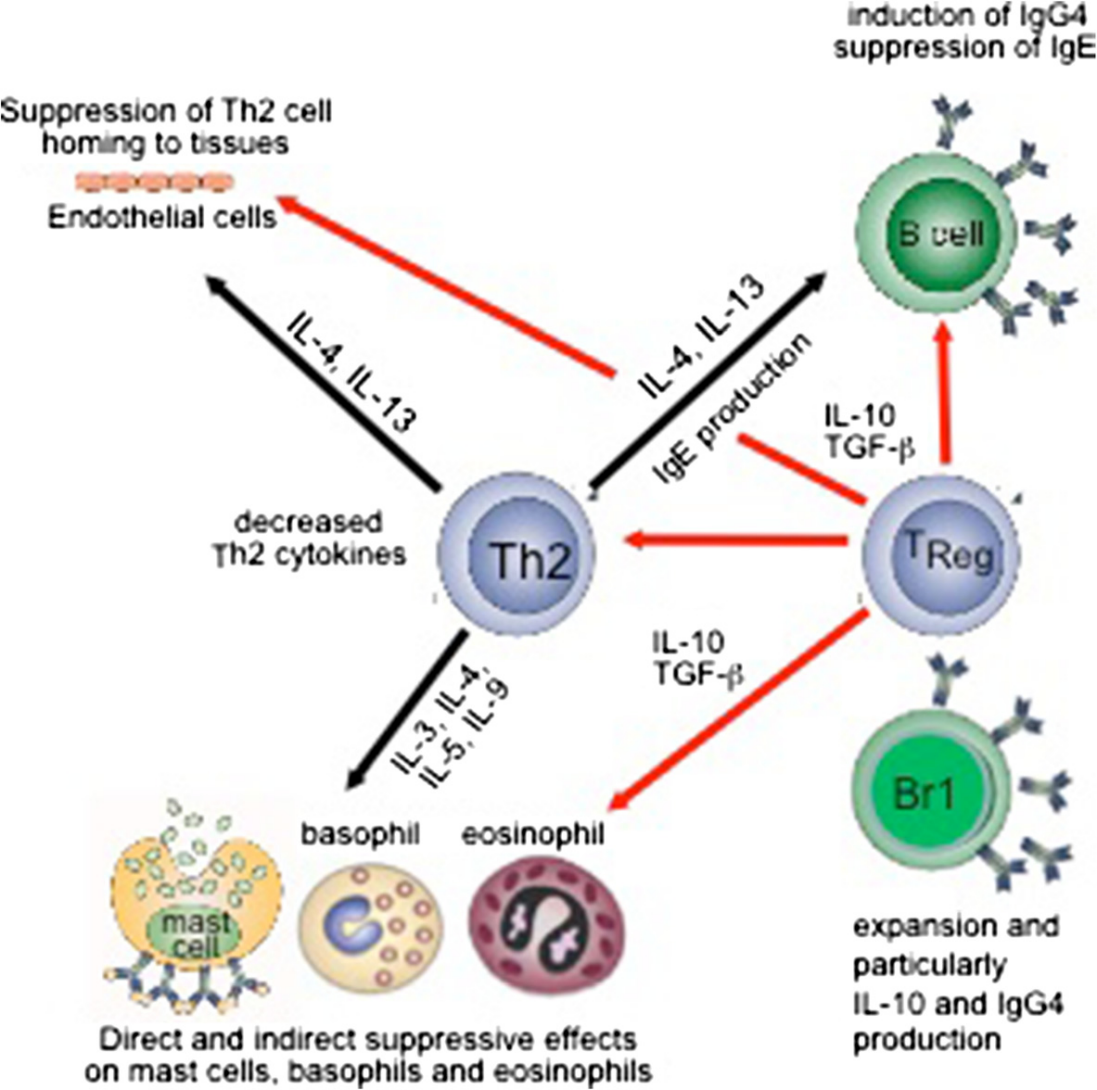
**Role of Treg and Breg cells in the suppression of allergic inflammation.** The balance between Th2 cells and Treg cells is decisive for the development or suppression of allergic inflammation. Treg cells and their cytokines suppress Th2 type immune responses and contribute to the control of allergic diseases in several major ways. Red arrows indicate the regulatory and suppressive effects of Treg cells, which exert their regulatory functions directly or indirectly on B cells by inducing IgG4 and IgA and suppressing IgE; on vascular endothelium by suppressing Th2 cell homing to tissues; on mast cells, basophils and eosinophils via direct and indirect suppressive effects; and on directly and indirectly suppression of epithelial cell activation and proinflammatory properties. In addition, B reg cells also suppress effector T cells and contribute to IgG4 synthesis.

The induction of a tolerant state in peripheral T cells represents an essential step in AIT (Figure 2). Peripheral T cell tolerance is characterized mainly by generation of allergen-specific Treg cells [130-132] and decrease in Th2 and Th1 cells [133]. It is initiated by IL-10 and TGF-β, which are increasingly produced by the antigen-specific Treg cells [130-132,134]. Subsets of Treg cells with distinct phenotypes and mechanisms of action include the naturally occurring, thymic selected CD4^+^CD25^+^ Treg cells, and the inducible type 1 Treg cells (Tr1) (Figure 1) [135]. Different studies show roles for both subsets suggesting an overlap in particularly the inducible subsets of Treg cells in humans. Their first effect is realized by suppression of allergen-specific Th2 and Th1 cells. The suppression by these cells could partially be blocked by the use of neutralizing antibodies against secreted or membrane-bound IL-10 and TGF-β. In coherence with this, it has been shown that CD4^+^CD25^+^ Treg cells from atopic donors have a reduced capability to suppress the proliferation of CD4^+^CD25^−^ T cells [136]. The presence of local Foxp3^+^CD25^+^CD3^+^ cells in the nasal mucosa, their increased numbers after immunotherapy, and their association with clinical efficacy and suppression of seasonal allergic inflammation strengthen the concept of allergen tolerance based on Treg cells in humans [137]. These findings were coined by tracking specific T cells with allergen class-II tetramers: clinical tolerance induction in humans is associated with a marked loss of IL-4-producing T-cells and the acquisition of IL-10-producing and FOXP3-positive antigen-specific CD4^+^ T-cells [138]. In addition to conventional immunotherapy, peptide immunotherapy in allergic asthma generates IL-10-dependent immunological tolerance associated with linked epitope suppression. Treatment with selected epitopes from a single allergen resulted in suppression of responses to other (“linked”) epitopes within the same molecule [139]. Treg cells and suppression of allergen-specific immune response in the course of AIT has been shown in many different AITs [140]. Similar findings of induction of IL-10 and Treg cells have been observed in mouse models of AIT only when prolonged schedules are used [141].

IL-10-producing antigen-presenting cells, such as B cells [142] and dendritic cells [123] as well as clonally expanded IL-10-producing allergen-specific Tr1 cells [131,143] all contribute to the suppressive effects of IL-10 in different models. IL-10 suppresses T cells by blocking CD2, CD28 and inducible co-stimulator (ICOS) co-stimulatory signals in a rapid signal transduction cascade [144]. In the presence of IL-10, a direct inhibition on CD2, CD28 and ICOS signaling in T cells occurs via utilization of Src-homology-2 domain containing tyrosine phosphatase (SHP-1) by IL-10 [144,145]. SHP-1 rapidly binds to CD28 and ICOS and dephosphorylates them [144]. Supporting these findings, spleen cells from SHP-1-deficient mice show increased proliferation with CD2, CD28 and ICOS stimulation in comparison to wild-type mice, which was not suppressed by IL-10. Generation of dominant negative SHP-1-overexpressing T cells or silencing of the SHP-1 gene by small inhibitory RNA (siRNA) both altered SHP-1 functions and abolished the suppressive effect of IL-10 [144-146]. Interestingly, the suppressive effect of IL-10 was not observed in other IL-10 family cytokines IL-19, IL-20, IL-22, IL-24 [147]. In addition to T cells, IL-10 also exerts inhibitory effect on activated monocytes and macrophages [148]. It has been shown in monocytes and DC that IL-10 suppresses co-stimulatory molecules and down regulates MHC class-II molecules and APC capacity [149]. Furthermore, IL-10 induces the expression of the suppressor of the cytokine-signalling-3 (SOCS3) gene that might play a role in the inhibition of the IFN-γ-induced tyrosine phosphorylation of Stat1 [150].

TGF-β is essential for the maintenance of immunological self-tolerance [151]. TGF-β induces the conversion of naive CD4^+^CD25^−^ T cells into CD4^+^CD25^+^ T cells by the induction of FoxP3 [152], and TGF-β signaling is required for *in vivo* expansion and immunosuppressive capacity of CD4^+^CD25^+^ T cells [153]. In addition, RUNX1 and RUNX3 transcription factors play an essential role in FOXP3 development both in humans and mice [154]. However, the exact suppressive mechanisms behind TGF-β activation of Smad pathways remain to be elucidated.

#### Treg and Breg cells in healthy immune response to allergens in high dose exposed individuals

Two high dose allergen exposure models have been studied in humans. These are immune response to bee venom allergens in bee keepers and immune response to cat allergens in cat owners [135,155]. If a detectable immune response is mounted, Tr1 cells specific for common environmental allergens consistently represent the dominant subset in healthy individuals. They use multiple suppressive mechanisms, IL-10 and TGF-β as secreted cytokines, and cytotoxic T lymphocyte antigen 4 and programmed death 1 as surface molecules. Healthy and allergic individuals exhibit all three, i.e. Th1, Th2, Tr1 type allergen-specific subsets in different proportions [143]. Accordingly, a change in the dominant subset and the balance between Th2 and Treg cells may lead to either allergy development or recovery.

It was found in allergic children that Treg cells increase during pollen season [156]. Whether these CD4^+^CD25^high^ T cells directly contribute to inflammation or their increased levels keep the inflammation at low levels remains as an important research question. Circulating allergen-specific CD4^+^CD25^high^Foxp3^+^ T-regulatory cells do not show a major difference between nonatopic and atopic individuals [157]. However, it was demonstrated that FOXP3 expression shows a negative correlation with IgE, eosinophilia and IFN-γ levels and FOXP3^+^/CD4^+^ ratio is significantly low in asthma and atopic dermatitis [158]. CD4^+^CD25^+^ Treg cells have been associated with the spontaneous remission of Cow’s milk allergy. Children who outgrew their allergy (tolerant children) had higher frequencies of circulating CD4^+^CD25^+^ T cells and decreased *in vitro* proliferative responses to bovine beta-lactoglobulin in peripheral blood mononuclear cells compared with children who maintained clinically active allergy [159]. Peripheral tolerance utilizes multiple mechanisms to suppress allergic inflammation. Treg cells contribute to the control of allergen-specific immune responses by a)Suppression of antigen-presenting cells that support the generation of effector T cells; b) suppression of Th2 and Th1 cells; c) suppression of allergen-specific IgE and induction of IgG4; d) suppression of mast cells, basophils and eosinophils; e) interaction with resident tissue cells and remodeling [135]. In addition to immune suppression, decrease in antigen-specific Th2 repertoire because of central lymphatic organ homing or deletion my play a role in the mechanisms of allergen tolerance [160,161]. In some cases and types of immunotherapies, the suppression of Th2 cells was found to be transient [162].

Allergen tolerance in healthy individuals can be broken under certain conditions. In a recent study, human tonsils were studied that contain allergen-specific T cells but show very low levels of allergen-induced T-cell proliferation, thus representing a very suitable *in vivo* model to assess mechanisms of breaking allergen-specific T-cell tolerance [163,164]. It was demonstrated that triggering of Toll-like receptor (TLR) 4 or TLR8 and the proinflammatory cytokines IL-1beta or IL-6 break allergen-specific T-cell tolerance in human tonsils and peripheral blood through a mechanism dependent on the adaptor molecule myeloid differentiation primary response gene 88. In particular, myeloid DCs and stimulations that activate them broke the tolerance of allergen-specific CD4 T cells, whereas plasmacytoid DCs and stimulations that activate them, such as TLR7 and TLR9, did not have any effect. Tolerance-breaking conditions induced by different molecular mechanisms were associated with a mixed cytokine profile with a tendency toward increased levels of IL-13 and IL-17, which are T2 and T17 cytokines, respectively. These findings suggest that certain innate immune response signals and proinflammatory cytokines break allergen-specific CD4 T-cell tolerance in healthy subjects, which might lead to the development or exacerbation of allergic diseases after encountering microbes or inflammatory conditions [163]. Viral infections represent important candidates for breaking of allergen tolerance, because as a virus infected lymphoid tissue, human tonsillar cytokine expression is closely related to existing viral infections and shows distinct clusters between antiviral and immune regulatory genes [165].

In addition to Treg cells, IL-10-producing regulatory B cells suppress immune responses, and lack of these cells leads to exacerbated symptoms in mouse models of chronic inflammation, transplantation, and chronic infection [166]. In a recent study human inducible IL-10-secreting B regulatory 1 (BR1) cells were characterized. Human IL-10+ BR1 cells expressed high surface CD25 and CD71 and low CD73 levels. Sorting of CD73-CD25 + CD71+ B cells allowed enrichment of human BR1 cells, which produced high levels of IL-10 and potently suppressed antigen-specific CD4+ T-cell proliferation [166]. IgG4 was selectively confined to human BR1 cells. B cells specific for the major bee venom allergen PLA isolated from nonallergic beekeepers show increased expression of IL-10 and IgG4. Furthermore, the frequency of IL-10+ PLA-specific B cells increased in allergic patients receiving allergen-specific immunotherapy. This study demonstrates two essential *in vivo* evidence for allergen tolerance: the suppressive B cells and IgG4-expressing B cells that are confined to IL-10+ BR1 cells in human subjects [166]. It was recently demonstrated that solely IL-10-overexpressing B cells acquired a prominent immunoregulatory profile comprising upregulation of suppressor of cytokine signaling 3 (SOCS3), glycoprotein A repetitions predominant (GARP), the IL-2 receptor alpha chain (CD25), and programmed cell death 1 ligand 1 (PD-L1) [167]. These cells showed a significant reduction in levels of proinflammatory cytokines (TNF-alpha, IL-8, and macrophage inflammatory protein 1alpha) and augmented the production of anti-inflammatory IL-1 receptor antagonist and vascular endothelial growth factor. Furthermore, IL-10-overexpressing B cells secreted less IgE and potently suppressed proinflammatory cytokines in PBMCs, maturation of monocyte-derived dendritic cells (rendering their profile to regulatory phenotype), and antigen-specific proliferation [167].

#### Modulation of allergen-specific IgE and IgG responses during AIT

Peripheral T cell tolerance is rapidly induced during AIT, however there is no evidence for B cell tolerance in the early course [130]. AIT induces a transient increase in serum specific IgE followed by gradual decrease over months or years of treatment (Figure 1) [168,169]. In pollen-sensitive patients, desensitization prevents elevation of the serum specific IgE during the pollen season [170]. The changes in IgE levels cannot explain the diminished responsiveness to specific allergen due to AIT, since the decrease in serum IgE is relatively late and does not correlate with clinical improvement after AIT.

Subclasses of IgG antibodies, especially IgG4 is thought to capture the allergen before reaching the effector cell-bound IgE, and thus to prevent the activation of mast cells and basophils. IgG4 antibodies can be viewed as a marker of introduced allergen dose and they have the ability to modulate the immune response to allergen. However, the relationship between the efficacy of AIT and the induction of allergen-specific IgG subgroups remains a controversial issue with serum concentrations of allergen-specific IgG correlating with clinical improvement in some studies, but not in others [171,172]. Allergen-specific IgG may be directed against the same epitopes as IgE, resulting in direct competition for allergen binding and a “blocking” effect. The concept of blocking antibodies has been revaluated. Analysis of the IgG subtypes induced by AIT has shown specific increases in IgG1 and particularly IgG4, with levels increasing 10-100-fold [173,174]. There is accumulating evidence that specific immunotherapy also influences the blocking activity on IgE-mediated responses by IgG4. Results suggest that successful specific immunotherapy is associated with an increase in IgG blocking activity that is not solely dependent on the quantity of IgG antibodies [175,176]. In a recent study, inhibition by IgG required Fcγ receptor-IIB. One IgG against a single epitope on the major allergen was able to block the degranulation of basophils from individuals with cat allergy. The inhibitory potential of IgG antibodies increased when larger allergen-IgG complexes were formed. It seems to be relevant rather to measure the blocking activity and or affinity of allergen-specific IgG or IgG subsets, particularly IgG4 and also IgG1 instead of their levels in sera [177].

There are several features of IgG4, which may play a role in its non inflammatory role. IgG4 hinge region has unique structural features that result in a lower affinity for certain Fcγ receptors and the ability to separate and repair by dynamic Fab arm exchange leads to bi-specific antibodies that are functionally monomeric [178,179]. Furthermore, IgG4 does not fix complement and is capable of inhibiting immune-complex formation by other isotypes, giving this isotype anti-inflammatory characteristics. In a clinical trial with five recombinant *Phleum* allergen mixures, all treated subjects developed very strong allergen-specific IgG4 and also increased IgG1 antibody responses. Some patients who were not initially sensitized to Phl p 5, for example, developed strong specific IgG4, but not IgE antibody responses specifically against that allergen [173]. This demonstrates that extract based antibody measurements may provide a wrong information and studies on mechanisms of AIT should be performed with single allergens.

It is highly possible that the decrease in IgE/IgG4 ratio during AIT is a feature of skew from allergen-specific Th2 to Treg cell predominance. IL-10 is a potent suppressor of both total and allergen-specific IgE, while it simultaneously increases IgG4 production [131,180]. Thus, IL-10 not only generates tolerance in T cells; it also regulates specific isotype formation towards a non-inflammatory phenotype. The healthy immune response to Der p 1 is associated with increased specific IgA and IgG4, small amounts of IgG1 and almost undetectable IgE antibodies in serum [132]. In the same study house dust mite-AIT did not significantly change specific IgE levels after 70 days of treatment; however, a significant increase in specific IgA, IgG1 and IgG4 was observed [132]. The reason for the long-time gap between the change in T cell subsets, but not IgE levels is not easily explainable by the half-life of this antibody. In this context, the role of bone marrow-residing IgE-producing plasma cells with very long life-span remain to be investigated [181].

## Conclusion

During the past 20 years, major advances have been made in understanding the molecular and cellular mechanisms of allergen tolerance in humans. The demonstration of allergen-specific T and B cell tolerance, particularly that mediated by the immune-suppressive functions of IL-10, led to a major conceptual change in this area [182]. AIT has multiple mechanisms of action with the involvement of many cell subsets. These effects comprise *very early effects related to antigen-presenting cells and adjuvants,* desensitization of effector cells, antigen-specific immune tolerance in T and B cells and regulation of IgE and IgG4 (Figure 1). Similar mechanisms are observed in high dose allergen tolerance in healthy bee keepers and non allergic cat owners. The kinetics and intensity of these events change according to the type of AIT vaccine that is used and the place of administration.
